# Diabetes in COVID-19 patients: challenges and possible management strategies

**DOI:** 10.1186/s43168-021-00099-2

**Published:** 2021-12-04

**Authors:** Riyan Al Islam Reshad, Sumaiya Hafiz Riana, Mohammad Al-baruni Chowdhury, Abu Tayab Moin, Faruque Miah, Bishajit Sarkar, Nurnabi Azad Jewel

**Affiliations:** 1 Department of Genetic Engineering and Biotechnology, Faculty of Life Sciences, Shahjalal University of Science and Technology, Sylhet, Bangladesh; 2 Department of Genetic Engineering and Biotechnology, Faculty of Biological Sciences, University of Chittagong, Chattogram, Bangladesh; 3 Department of Biotechnology and Genetic Engineering, Faculty of Biological Sciences, Jahangirnagar University, Dhaka, Bangladesh

**Keywords:** Diabetes mellitus (DM), COVID-19, ACE2

## Abstract

**Background:**

The recent pandemic of coronavirus disease 19 (COVID-19) has been causing intense stress among the global population. In the case of hospitalized and ICU-admitted COVID-19 patients with comorbidities, it has been observed that a major portion of them are diabetic. Therefore, researchers had indicated a link between diabetes mellitus (DM) and COVID-19. Furthermore, DM is a potential risk factor for the severity of COVID-19 cases. Thus, in this study, the correlation existing between diabetic patients and COVID-19 was summarized.

**Main body of the abstract:**

Diabetic patients have a weaker immune system, less viral clearance rate, malfunctions of metabolic activity due to their high blood glucose level, and other associated problems. This does not increase the susceptibility for the patients to be infected with COVID-19. However, the severity of COVID-19 can worsen due to the comorbidity of DM.

**Short conclusion:**

Proper management, appropriate use of drugs that do not increase the ACE2 expression, lowering blood glucose level, decreasing the susceptibility of SARS-CoV-2, and maintaining a healthy lifestyle could be effective.

## Background

The SARS-CoV-2 (severe acute respiratory syndrome; for this, epidemiological findings, clinical manifestation, signs and symptoms, risk factors, management, and treatment were analyzed to conclude coronavirus 2) virus first emerged in Wuhan, China, back in December of 2019, which then started to spread in many countries from its site of origin [1]. In March of 2020, WHO (World Health Organization) named the disease COVID-19 (coronavirus disease 19) caused by highly contagious SARS-CoV-2 and declared the outbreak as a global pandemic. Since then, this respiratory disease-causing virus was reported to spread across more than 188 countries, where approximately 157,690,329 cases were confirmed till May (source: Johns Hopkins University Coronavirus Resource Centre: https://coronavirus.jhu.edu/map.html). This is the third time a virus from the *Coronaviridae* family has been transmitted from animal sources and caused respiratory-related disease in the human population. The first coronavirus-related outbreak called SARS (severe acute respiratory syndrome) was caused by SARS-CoV (severe acute respiratory syndrome-related coronavirus) during 2002–2004. Later, the second pandemic was caused by the MERS CoV virus, which was the pathogen for Middle Eastern respiratory syndrome (MERS) [2].

Based on the initial outbreak cases from China, researchers found a link between COVID-19 with comorbidities [3]. Deng and Peng reported that 25.1% had at least one underlying condition; concurrently hypertension (16.9%) and diabetes (8.2%) were two of the most prevalent comorbidities [4]. This correlated data between diabetes as a comorbidity for COVID-19 is concerning globally as diabetes has been considered the pandemic of the 21st century due to the escalation of DM in the older population and adolescents [5]. According to WHO, 2% of the global population, summing to approximately 65,666,000 people, were suffering from either form of DM (type 1 and type 2 DM) in the year 2016. Data from 187 WHO member states were analyzed to deduce DM as the 8th leading cause of disability-adjusted life-year (DALY) that jumped from the 15th rank of 2000’s DALY report [6]. Apart from the large size of the vulnerable diabetic community, the disease DM elevates the risk of harboring elevated symptoms or infection rate caused by SARS-CoV-2. Firstly, diabetic patients are highly susceptible to infection pathogens, and both DM1 and DM2 patients lie in risked groups of forming mucous membrane infection when exposed to causes [7]. Secondly, suppose diabetic patients are exposed and infected by SARS-CoV-2. In that case, there is a higher chance of a severe form of COVID-19 as diabetic patients are observed to have a lower rate of viral clearance and higher affinity of the pathogen to cellular binding [8]. Apart from the independent risk factors, certain drugs like glucagon-like peptide-1 receptor agonists may increase the angiotensin-converting enzyme 2 (ACE2) receptor numbers in organs like the liver, leading to the higher affinity of SARS-CoV-2 as the mentioned receptor is found to be responsible for the viral binding [9].

Therefore, DM as comorbidity is significant for the current COVID-19 pandemic as this large population is easily affected and prone to harbor severe symptoms of respiratory disease. In this context, simultaneous management of both conditions must be done for diabetic COVID-19 patients. In addition, drug adjustment must be done attentively as some of the DM-alleviating drugs can elevate the physical damages caused by COVID-19.

## Main text

### Epidemiological findings linking DM and COVID-19

As Zhou et al. indicated the risk of comorbidities in COVID-19 manifestation [3], Wu et al. have described DM as a particular risk factor for the severity and mortality of COVID-19 [10]. Despite the sample size of 24 subjects being a limitation for the study, Guo et al. deduced that diabetic COVID-19 patients without any other comorbidity were at risk for a severe case of pneumonia. In diabetic and nondiabetic subjects’ case comparison, diabetic COVID-19 patients had a higher level of most COVID-19–indicating biomarkers (e.g., D-dimers, serum ferritin, C-reactive protein, etc.) than the nondiabetic subjects, as well as higher mortality rate (16% vs. 0%). After the pandemic advanced, this supports the idea that DM impedes an individual’s innate immunity by reducing leukocytic phagocytosis and cell-mediated immunity [11]. Later in another retrospective study consisting of 1561 COVID-19 patients, Qiao et al. found 153 patients with DM as an underlying illness who suffered from a higher mortality rate (20.3% vs. 10.5%, *P* = 0.017) than age- and sex-matched nondiabetic COVID-19 patients.

Moreover, as a consequence, they required more intensive care indicating higher severity of COVID-19 in diabetics [12]. In another extensive multicentered cohort of 7.337 studies based in China, 13% of the COVID-19 patients had preexisting DM [13]. Four vital observations of this comparative study between diabetic and nondiabetic COVID-19 patients were (1) greater severity of COVID-19 symptoms (e.g., fatigue, cough, dyspnea, and fever) in diabetic patients, (2) frequency of other comorbidities in people with diabetes (e.g., coronary heart disease (CHD), cerebrovascular disease, chronic kidney disease, and hypertension), (3) higher incidences of COVID-19 biomarkers (e.g., C-reactive protein, procalcitonin, leukocyte, and coagulation status or D-dimer level), and (4) increased necessity of external oxygen supply in the form of invasive or noninvasive ventilation.

When SARS-CoV-2 spread from China to other countries, it particularly devastated the Italian and American populations. In a national report based on the pandemic scenario of Italy, 33% of comorbid COVID-19 patients suffered from DM [14]. While among COVID-19 patients from Lombardy (a region of Italy), 17% of 1591 subjects suffered from type 2 diabetes (T2D) [15]. In the USA case, DM is already a concern for the nation as approximately 10% of the population has one form of DM [16]. Three distinct cohort case studies have confirmed that DM (T1D or T2D) has deteriorated the COVID-19 condition in American diabetic patients. Centers for Disease Control and Prevention observed 24 subjects with COVID-19, among whom 14 were diabetic, leading to 58% having DM as comorbidity [17]. Although this small-scale study lacked confirmation of the severity among diabetic COVID-19 patients, Richardson et al. discovered the 3rd highest comorbidity was diabetes (33.8% or 1.808 subjects) among the observed cohort of 5700 COVID-19 patients. Among the patients who expired due to the severity of COVID-19, nondiabetic patients required less intensive care or artificial ventilation to suppress the symptoms than those with diabetes [18]. In a later observational study, 1122 adult COVID-19 patients were observed with diabetes and hyperglycemic condition. A total of 38.5% of the hospitalized patients were diabetic or hyperglycemic and faced a higher mortality rate than those without the conditions [19].

India has become the recent victim of COVID-19 as the third wave has led to a sharp incline in both confirmed cases and mortality rate. Before the recent surge, Mithal et al., among other researchers, also proved the link between DM and severity of COVID-19. They found 210 patients with either preexisting or new-onset DM among 401 COVID-19 patients suffered severe symptoms and required ICU admission [20], supporting the data found by Shi et al. [13]. Moreover, the correlation between the baseline Hba1c and severity score of COVID-19 was significant in the Indian and higher mortality rates.

As the first country with the outbreak and 3 of the countries with a high prevalence of COVID-19 epidemiological data from China, Italy, the USA, and India have shown the noteworthy link between COVID-19 manifestation among the population previously suffering from DM.

### Clinical manifestations of COVID-19 patients with diabetes

Studies have shown that the presence of glucagon plays a vital role in endogenous hyperglycemia. Insulin on the liver directly resists hepatic glucose production. Therefore, excess glucagon helps with hyperglycemic effects [21]. Mitochondria are the principal source of reactive oxygen species (ROS). A study showed that hyperglycemia-induced mitochondrial ROS production might be a key player in developing diabetes-related complications. Also, mitochondrial ROS might play a vital role behind type 2 diabetes resisting insulin [22]. Insulin is secreted in a two-phase pattern. Beta cell abnormalities in type 2 diabetes patients are found in the first phase [23]. Studies suggest that beta cell decline starts about 12 years before diagnosis [24]. Though the mechanism behind beta cell degradation is unclear, genetic factors [25], environmental factors, and hyperglycemia and hyperlipidemia can be the main reasons behind beta cell declining [26]. The SARS-CoV-2 virus probably binds with epithelial cells of the nasal cavity and starts the replication process. ACE2 works as the main receptor for SARS-CoV-2. The virus multiplies and travels through the respiratory tract, thus triggering the immune system. Infected epithelial cells are the main source of beta and lambda interferons. CXCL10, which is an immune responsive cytokine, is responsible for the immune responses against SARS-CoV-2. In severe cases, this affects pulmonary infiltrates, causing fatal diseases. Though the fatality rate is 2%, it differs with age. The virus mostly affects type 2 alveolar cells rather than type 1 cells. The virus replicates within the type 2 cell, thus eventually destroying the cells. Older people generally have decreased mucociliary clearance, enabling the virus to increase more rapidly [27]. In nonhuman primate models, it was found that the amount of the virus rapidly advances throughout the respiratory system. Replication in the upper respiratory tract transmits the virus between hosts, and if replication occurs in the lower respiratory tract, it causes lung disease [28]. The pathogenesis of COVID-19 as a disease targeting the respiratory system was pneumonia and RNAaemia, associated with cardiac injury. High blood levels of cytokines were also noticed in patients with COVID-19, including IL1-β, IL1RA, IL7, IL8, IL9, IL10, and other pro-inflammatory cytokines, which are believed to be the reason for increasing severity [29].

### Possible mechanisms of diabetes worsening COVID-19 progression

Diabetes has been a major cause of severity and death in previous viral pandemics like influenza (H1N1), SARS-CoV, and MERS-CoV. Though some studies did not find any connection between COVID-19 and diabetes, some reports from China and Italy showed patients with diabetes had more mortality and severity rates [30]. In addition, some data shows COVID-19 in patients with diabetes might be affected by releasing hyperglycemic hormones, resulting in higher levels of glucose and abnormal glucose variability [31]. Hyperglycemia leads to aggressive glycosylation (disturbance in determining structure, features, and stability of protein) that causes failure in receptor signaling and disrupts the functions of immunoglobulins. This glycosylation disturbance of IgG may cause susceptibility to COVID-19. As a result, these patients are more likely to require mechanical ventilation and admission to ICU with higher mortality [32, 33].

**Fig. 1:**
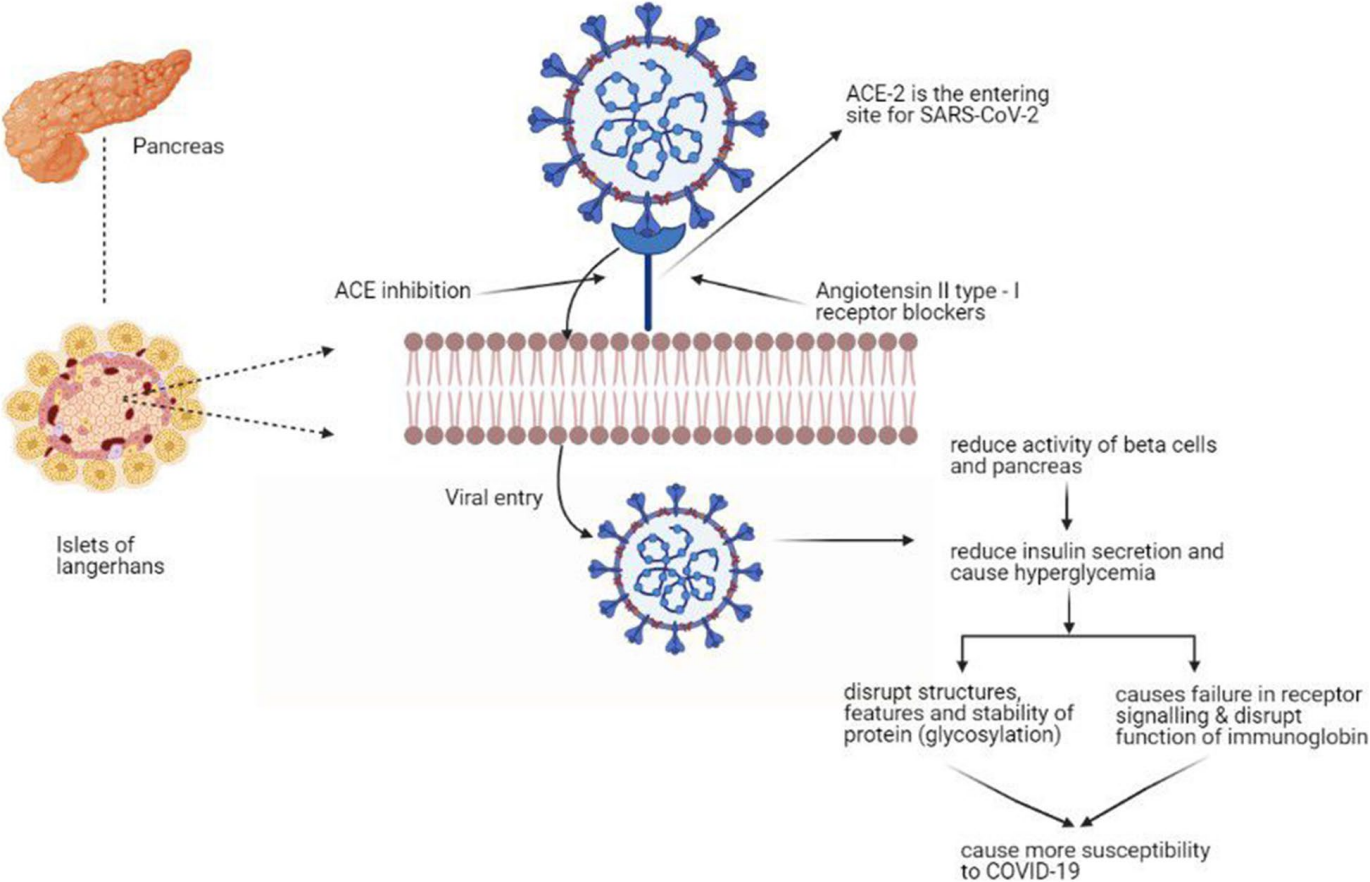
Mechanisms of diabetes worsening COVID-19 progression

Again, people with diabetes generally suffer from low-grade chronic inflammation, which possibly triggers cytokine storms. This also appeared to be one of the significant causes of the severe cases of COVID-19 cases of pneumonia and death of many patients. Severe pneumonia can lead to severe hypoxia, respiratory failure, multi-organ failure, shock, and death [33]. ACE2 acts as the entering site for SARS-CoV-2 to enter into the human body. ACE2 is generally found in the liver and endocrine pancreas with a possible role in developing insulin resistance and reducing insulin secretion. So, there are possibilities that SARS-CoV-2 can affect both pancreas and beta cells, worsening hyperglycemia during acute infection [34]. Another study reports that diabetes has a connection in activating the renin–angiotensin system in different tissue systems. Also, people with type 1 or type 2 diabetes are often treated with ACE inhibitors, and angiotensin II type I receptor blockers (ARBs) might increase the expression of ACE2. And this increase in the expression of ACE2 can augment the risk of viral infections. The observation also suggests that switching people to other agents from renin–angiotensin system blockers might modify the risk [35]. A Chinese study was conducted on 39 COVID-19 patients compared with 39 healthy siblings. The outcome showed that 20 of the 39 patients developed diabetes during hospitalization. As immunostaining for ACE2 is widely spread in pancreatic islets, it was suggested that SARS-CoV-2 might have damaged the islet causing insulin-dependent DM [36]. Though many reports indicate little or no connection between COVID-19 and diabetes, plenty of studies suggest a connection between the diseases (Fig. 1).

### Risk factor and management of diabetic patients during COVID-19 pandemic

#### Risk factor of diabetic patients

##### Obesity

It was known that individuals having diabetes are at the potential risk for various infectious diseases and case severity. At the SARS outbreak, it was observed that the rate of ICU admission, the need for mechanical ventilation, and mortality was 3.1-fold greater in diabetic patients than nondiabetic patients [37].

##### Aggravated inflammatory storm

Diabetic patients generally suffer from low-grade chronic inflammation, which might lead them to excessive cytokine storms, the severe outcome of COVID-19, and elevated mortality [34, 38]. Some recent studies on biochemical features of COVID-19 diabetic patients and nondiabetic patients revealed that lymphocyte count is significantly lower. In contrast, the count of neutrophils is noticeably higher than nondiabetic patients [32, 39, 40]. Besides, neutrophil to lymphocyte ratio (NLR), high-sensitivity C-reactive protein, and some inflammation-related biomarkers such as interleukin (IL)-6 are found to be more elevated in diabetic patients [32, 41]. Moreover, a significant increase in serum ferritin accelerates the activation of the monocyte–macrophage system, which can be a crucial part of inflammatory storms [40].

##### Immunodeficiency

In diabetic patients, it is seen that besides elevated NLR levels, the total amount of T cell, CD4^+^, and CD8^+^ T cell are significantly reduced in a severe case of COVID-19 patients with the unregulated immune response [42, 43]. The nonspecific and first-acting innate immune defenses that provide initial host response are obstructed and not working properly in people with diabetes.

Neutrophils, the most abundant white blood cell in circulation, play a major role in clearing infection through digesting, blocking, and disabling the invading foreign particles entering the body. But, in diabetic patients, it shows an abnormality in its function, which includes migration towards the inflammatory site, lytic protease release, phagocytosis, apoptosis, and reactive oxygen species production [44]. Besides, patients with diabetes have the oversecretion of tumor necrosis factor (TNF)-α, interleukin (IL)-1β, and IL-8, which increased the susceptibility for the various pathogens to attack [45]. Moreover, M1 pro-inflammatory macrophages release inflammatory mediators who have a role in local and systemic inflammations also found an elevated level than actual [46].

Natural killer cells (NK cells) play a major role in killing the cells infected with the various pathogens or present in stress conditions expressing major histocompatibility complex (MHC). In diabetic patients, the phenotype and functional activity of NK cells found change which decreases the viral recognition ability and hampers the activation of NK and CK8^+^ T lymphocytes receptors [47].

To maintain pro-inflammatory and anti-inflammatory damage, CD4^+^ effector T cell plays a vital role and controls host immunity and damage of inflammations [48], but in type 2 diabetic patients, and the inflammatory process is promoted [49, 50].

Further investigations have revealed that in adipose tissue of diabetic patients, anti-inflammatory macrophages are transformed into pro-inflammatory macrophages, and Tregs are converted into helper Th1 and Th17 CD4^+^ T cells [51, 52]. In detail, pro-inflammatory macrophages Th1 responsible for cell-mediated immunity and phagocytic inflammation are upregulated [53]. Besides, another pro-inflammatory and CD4^+^ T cell subtype, Th17, which secrete IL-17 and stimulates TNF-α, also increases [54]. Moreover, Treg cell expression is reduced in inpatient with people with diabetes responsible for suppressing Th1, Th2, & Th17 responses and improving insulin resistance (IR) and inflammatory response [55].

#### Increased ACE2 expression

It is revealed that SARS-CoV-2 uses the ACE2 receptors to facilitate cellular entry, and this is also responsible for the elevated level of transmission rate from one person to another [56]. Besides, research has found that SARS-CoV-2 has a much higher binding affinity with the ACE2 receptors than the previously isolated SARS-CoV, which is approximately 10- to 20-fold higher [57]. ACE2 expression has been observed in multiple organs such as the liver, kidney, hearts, testis, lungs, pancreas, bladder, stomach, ileum, etc. [58, 59]. Moreover, researchers have found the overexpression of ACE2 in circulation in diabetic patients, which may increase the chance of viral infection. It is seen that the administration of insulin has significantly decreased the expression level of ACE2 [60–62]. In types 1 and 2 diabetic patients, ACE2 and enzymatic activity are found at elevated urine levels [63, 64]. Besides, the blood glucose level and the presence of ACE2 in urine are positively controlled [65]. However, ACE2 expression in kidney tissue is downregulated in diabetic patients [66]. Thus, ACE2 expression can vary depending on the tissue. Researchers have revealed that increased ACE2 and angiotensin 1-7 play a protective effect against lung injury by vasodilatory and antifibrotic effects, whereas reduced ACE2 level may predispose to more severe lung injury [67, 68].

So the diabetes-induced expression of ACE2 may cause case severity and a risk factor for COVID-19 in diabetic patients.

#### Uncontrolled glycemic status

Patients with diabetes with unregulated blood glucose levels are at higher risk of case severity and observed to have a higher number of hospital admission and acquired pneumonia [69–71]. In hyperglycemic states, susceptibility for the pathogen to attack increases, the interleukin production response to the pathogen decreases, and the activity of phagocytosis and polymorphonuclear leukocytes reduces [72]. Besides, individuals with type 1 diabetes are at higher risk of developing diabetic ketoacidosis (DKA) when acquiring an infection. Moreover, reports have found increased levels of glucose secretions through airways [73]. This may be because of the pulmonary structural change in people with diabetes [74]. The ACE2 receptors are also found expressed in pancreatic beta cells [75]. So, the entry of this virus can damage the pancreatic beta cell and trigger the glucose level in blood and lead to hyperglycemic conditions [36].

#### Coagulations

COVID-19 is associated with elevated coagulation activity [76]. Intra-vessel coagulation can occur due to the endothelial dysfunction which is associated with hypoxia. Diabetic patients are reported with a prothrombotic state, in which an imbalance between clotting factors and fibrinolysis accelerates the risk of thromboembolic events [77]. In a Chinese study, longer prothrombin time and a high concentration of D-dimer presence were found in the non-survivor patients with diabetes [32].

Diabetic patients face many internal body complications such as inflammatory storm, hyperglycemic state, coagulation possibilities, increased level of ACE2 receptor, immunodeficiency, etc.. Besides, they have other diseases such as high blood pressure, cardiovascular disease, renal problem, vision problem, and so many. All these things do not increase the risk of getting infected with SARS-CoV-2. But once they get infected, the case becomes so severe that the mortality rate is elevated. So, they should maintain proper precautions not to be infected, and if they get infected, proper medication and a healthy lifestyle should lead to consulting with the doctor.

### Management of diabetic patients

The top priority for diabetic patients is to maintain their blood glucose levels. It has already been known that in the case of COVID-19, unbalanced blood glucose level may lead to higher susceptibility for the pathogen, a higher rate of hospital admission, and an elevated level of mortality [78]. Therefore, after hospitalizations, the patients’ blood glucose level frequently needs to be monitored and proper insulin dosage should be maintained properly. Moreover, mechanical ventilation may be needed for these patients in case of respiratory distress, but it may also increase the aerosolization of the virus [79].

For type 2 diabetics, metformin is considered the first-line treatment option in these pandemic situations. It generally activates the AMP-activated protein kinase in the liver [80]. This drug was first developed as an anti-influenza drug, but tolerance to glucose-lowering activity was a side effect [81]. Metformin activates AMPK and leads to functional changes in the ACE2 receptors by phosphorylation [82]. So, such a kind of alteration can change the binding of coronavirus with its ACE2 receptors. It was previously known that this virus enters the host cell through ACE2 receptors and is imbalanced in the renin–angiotensin system. In contrast, metformin prevents this condition by activating ACE2 through AMPK signaling pathways [83]. So, there is no contradiction in taking metformin if a diabetic patient is infected with COVID-19; rather, it may be beneficial.

There are some antidiabetic drugs responsible for controlling blood glucose levels, such as glucagon-like peptide-1 receptor agonists (GLP-1Ra), sodium–glucose transporter 2 (SGT-2) inhibitors, and dipeptidyl peptidase 4 (DPP4) [84–86]. The GLP-1 and SGT-2 drugs have some additional benefits of anti-cardiovascular and anti-kidney diseases. This may be beneficial during pandemics as cardiovascular and kidney diseases are reported for the worse prognosis of COVID-19 [87]. Moreover, anti-inflammatory and anti-adipogenic effects with insulin resistance antagonism are reported in GLP-1Ra [88]. However, both GLP1-Ra and SGLT-2 inhibitors may elevate the amount of ACE2 and create overexpression [89]. Thus, this drug has much more serious consequences if individuals with diabetes are infected with COVID-19. On the other hand, DPP4 is generally used for type 2 diabetes targeting the incretin system [90]. Recently, some reports demonstrate that diabetic patients administered with DPP4 may decrease the case severity [91].

Thiazolidinediones (TZDs) is a commonly used drug for type 2 diabetes that treats insulin resistance [92]. Pioglitazone, a TZD-type drug, has been found to inhibit the secretion of pro-inflammatory cytokines IL-6 [93]. Pro-inflammatory states are considered to have a worse prognosis of COVID-19. Thus, this type of drug is considered to be supportive therapy for COVID-19 [94].

ACE inhibitors (ACEI) and angiotensin-receptor blockers (ARBs) are generally anti-hypersensitive and reno-protective drugs, respectively, and prescribed to individuals with diabetes [95, 96]. They increase the ACE2 expression, control hypertension, show anti-inflammatory effect, reduce injury in endothelial cells and organ fibrosis, maintain insulin sensitivity, and play a role in energy metabolism [97]. Besides, it also plays a role in lipid metabolism and normalizing coagulation cascade [98, 99]. Moreover, it has been reported that ARBs protect the epithelial cell of lung injury caused by pneumonia, influenza, and sepsis [100]. On the other hand, it has been reported that diabetic individuals taking ACE inhibitors are at increased risk of susceptibility to COVID-19 [101].

Corticosteroids (CS) play a role in the management of acute respiratory distress syndrome (ARDS) and sepsis, which is considered one of the management plans for COVID-19 [102]. CS is frequently linked to the development of hyperglycemia or diabetes mellitus (DM) [103]. The dosage of CS is one of the most worrying aspects of CS-induced diabetes. Higher CS doses (dexamethasone > 4 mg, hydrocortisone > 50 mg, prednisolone > 20 mg) can easily cause serious side effects [104]. According to a meta-analysis, the chances of CS-induced hyperglycemia and diabetes were 32.3 and 18.6%, respectively [105]. Several factors are linked to the onset of diabetes after the introduction of CS, including impairment of multiple pathways such as cell dysfunction [106], insulin resistance of tissues that can affect glucose metabolism [107, 108], reduction of peripheral glucose uptake by muscle and adipose tissues that raises blood glucose levels [107, 109], activation of genes involved in hepatic carbohydrate metabolism resulting in increased gluconeogenesis, and inhibition of genes involved in hepatic carbohydrate metabolism [110, 111]. Another treatment, type I interferon, was associated with bet cell damage and was responsible for worsening the blood glucose level [112, 113]. Thus, physicians should prescribe this medicine, remembering its worsening effect on the blood glucose level.

### Treatment for diabetic patients with COVID-19

Due to the ongoing quarantine, people face difficulties finding medicines, insulin, needles, glucose strips, etc.. Also, people have limited access to fresh fruits and vegetables; therefore, they tend to eat packaged foods high in calories, saturated fat, and trans-fat. All these factors cause glucose dysregulation, infections, hyperosmolar coma, ketoacidosis, and even acute cardiac events [114]. Patients with diabetes with developing symptoms of COVID-19 infection are at higher risk of adverse outcomes. Therefore, they need to be served with frequent glucose monitoring, a healthy diet, adequate hydration, and dose titration of glucose-lowering medication. Also, patients are recommended to take symptomatic therapy (i.e., paracetamol or acetaminophen), which reduces fever [115]. Diabetic patients, especially those treated with insulin, are at risk for developing hypoglycemia [116]. Therefore, regular monitoring of blood glucose is important. Continuous glucose monitoring (CGM) and flash glucose monitoring systems are useful and allow remote monitoring by healthcare providers. However, paracetamol may affect certain CGM sensors [117]. Nonsteroidal anti-inflammatory drugs (NSAIDs) such as Ibuprofen prevent fatal “cytokine storms,” in which the immune system of seriously ill patients can cause organ failure but increases SARS-CoV-2 activity [35] and slows recovery process [118]. However, the potential therapeutic options for COVID-19 in people with diabetes are discussed below with probable beneficial or hazardous effects.

#### Glucose lowering agents

Patients with severe SARS-CoV-2 develop acute respiratory distress syndrome (ARDS) due to dysregulated immune response, producing cytokine release syndrome (cytokine storm). Metformin, a first-line medication for treating type 2 diabetes, boosts immune responses and prevents ARDS compared with other anti-hyperglycemic agents. Still, it increases the risk of lactic acidosis and causes cardiovascular, renal, hepatic, and pulmonary disease [119]. Therefore, many health agencies have recommended avoiding sodium–glucose co-transporter 2 (SGLT2) inhibitors in COVID-19 patients and patients with a history of hypertension, type 2 diabetes mellitus (T2DM), and atherosclerotic cardiovascular disease such as heart failure [120]. Besides, treatment with glucagon-like peptide-1 receptor agonists (GLP-1RA) and SGLT2 plays a role in managing hyperglycemia and preventing heart damage. But it should not be used in patients with hemodynamic instability (unstable blood pressure) and renal and gastrointestinal dysfunction [121].

Moreover, DPP4 inhibitors reduce the risk of hypoglycemia and are relatively safe for renal functions. Adding a DPP4 inhibitor to basal insulin reduces plasma glucose concentrations without increasing the risk of hypoglycemia—even among hospitalized patients [122]. But these agents have less therapeutic benefit in patients with severe COVID-19. Furthermore, fine control of blood glucose is difficult using sulfonylureas. Also, the use of sulfonylureas and chloroquine increases the risk of hypoglycemia. So, they are recommended to be replaced with insulin.

Moreover, thiazolidinediones (e.g., pioglitazone) should not be used in patients with hemodynamic instability and hepatic or cardiac dysfunction because it causes fluid retention and edema, which may be seen in severe COVID-19 infection [123]. Insulin therapy is a preferred strategy to control blood sugar levels in hospitalized patients [124]. Insulin inhibits the synthesis of pro-inflammatory factors, producing fever, inflammation, tissue, and decreased chance of ARDS [125].

Therefore, patients with type 1 diabetes with COVID-19 and hyperglycemia may continue their insulin therapy, facilitating control of their blood glucose and ketone levels. Moreover, SGLT2 inhibitors may work as organ-protective agents in diabetic patients with COVID-19. However, further study is needed to ensure the use of DPP4 inhibitors in mild cases of COVID-19.

#### Immunomodulators

Immunomodulators can inhibit cytokines and treat the cytokine storm, essential in the pathogenesis of rapid deterioration and multi-organ dysfunction in patients with COVID-19. Moreover, chloroquine and hydroxychloroquine are now approved to treat inflammatory diseases due to low cost, but they are being tested for COVID-19 prevention, and treatment efficacy has been questioned [126]. However, chloroquine has demonstrated antiviral activity against five out of seven known human coronaviruses, including COVID-19 [127]. In addition, cytokines released to viral infections can induce the neuroendocrine system to release glucocorticoids and other peptides, impairing immune responses [128]. They are also used to treat severe acute respiratory distress syndrome (ARDS) and viral pneumonia, but their role in the treatment of COVID-19 is still investigated [129].

Furthermore, statins might inhibit the entry of SARS-CoV-2 into host cells by directly binding the main protease of the virus [130]. However, statins increase the expression of angiotensin-converting enzyme 2 (ACE2), the receptor for the virus. So, statins are often prescribed along with renin–angiotensin–aldosterone system (RAAS) blockers, in particular angiotensin-converting enzyme (ACE) inhibitors and angiotensin II receptor blockers (ARBs). Statins in hospitalized patients with COVID-19 reduce mortality and increase the recovery rate [131]. Hypertension, heart failure, and diabetic kidney diseases are treated with ACE inhibitors and ARBs. ACE inhibitors block ACE2 receptors, which may protect against COVID-19 infection [132].

Although immunomodulation therapies remain controversial in the COVID-19 context because they may negatively influence the immune response against SARS-CoV-2, several ongoing trials are trying to reduce overstimulation of the innate immune system. Further experiments are highly needed right away, especially on the effects of ACEI/ARBs and SGLT2 inhibitors in infected and severely ill patients.

In the presence of inflammatory stimuli, monocytes, macrophages, and dendritic cells generate the pro-inflammatory cytokine interleukin-1 (IL-1). In vitro, IL-1 causes -cell dysfunction and may play a role in diabetes aetiology [133–135]. A human recombinant IL-1 receptor antagonist (anakinra) improved glycemia in T2D patients for up to 39 weeks following therapy [136]. Patients with rheumatoid arthritis benefited from daily anakinra doses as well [137]. Furthermore, these trials have established the immunotherapeutic drug’s safety and tolerability profile.

In the late 1970s, cyclosporine A was one of the first immunosuppressive drugs used in people to improve kidney transplantation and prevent rejection [138]. In the biobreeding (BB) rat, this immunosuppressive medication prevented diabetes, and a pilot trial in 41 newly diagnosed T1D patients demonstrated its therapeutic prospects in humans [139, 140].

Vitamin D is required for Ca^2+^ metabolism and, as a result, for bone mineralization and mineral homeostasis. Under the effect of UV light in the skin, it can be produced endogenously [141]. Vitamin D supplementation might prevent or postpone T1D in people, according to birth cohort studies. Supplementing with vitamin D (50 g or 2000 IU per day) has been linked to a lower risk of developing T1D [142]. When children were treated with vitamin D early in infancy, the EURODIAB substudy found that they had a 33% lower risk of T1D [143].

#### Antiviral therapies

Antiviral therapies are used to stop the SARS-CoV-2 virus cell cycle and slow down disease progression. Several drugs, such as Ribavirin, interferon (IFN), favipiravir (FPV), and lopinavir (LPV)/ritonavir (RTV), have been used in patients with SARS or MERS. However, the efficacy of some drugs remains controversial [132]. Arbidol blocks the virus–cell membrane fusion by intercalating into membrane lipids. Wuhan data report shows better outcome in patients treated with Arbidol alone or in combination with other antiretroviral drugs [144]. Drugs that block viral RNA replication including lopinavir/ritonavir and darunavir—antiretrovirals for HIV—inhibit the viral protease [145]. The use of lopinavir/ritonavir (LPVr) in severe acute respiratory syndrome (SARS) indicated a favorable clinical response, but in SARS-CoV-2 infection, it shows limited efficacy. Remdesivir and favipiravir are viral RNA polymerase blockers that have been previously tested for Hepatitis C, Ebola, or Influenza, among others [120]. Remdesivir shortens the course of the COVID-19 disease but does not affect mortality [146]. Favipiravir inhibits RNA polymerase activity, but this drug is currently undergoing clinical trials in treating COVID-19. Also, the use of classical anticoagulant therapies such as heparin was initially applied to COVID-19 patients at risk of thrombotic and thromboembolic events [147].

Generally, there are no verified antivirals to date specific to COVID-19. However, several are used based on limited clinical data and tested in clinical trials. If their efficiency is proved, they could be used in patients with diabetes, although caution should always be maintained properly.

### General precautions and future guidelines for the patient of COVID-19 with diabetes

#### Precautions

Prevention is always better than cure. Therefore, people who have diabetes should take preventive steps and attempt to safeguard themselves from COVID-19 infection in the following ways:

i. **Maintaining rules of hygiene**
  Washing hands frequently with soap and water. Alcohol-based sanitizer is also preferred.
  Cleaning contaminated surfaces such as tabletops, doorknobs, railings, countertops with disinfectant. These surfaces can be contaminated by the touch of a COVID-19–infected person [148].
  Avoiding the habit of touching eyes, mouth, nose, and unwashed hands
  Touching surfaces should be eluded that are touched by others
  Covering mouth and nose with elbow or tissue and disposing of tissue after using one time
ii. **Practicing social distancing**
  Tiny droplets conduct the spread of novel coronavirus sprayed into the air when an infected person sneezes or coughs. If anyone surrounds within 6 feet/ 2 m of an infected person, he may inhale it. So, maintaining a safe distance of about 6 feet in public and working places is better prevention **[**149].
  Avoiding any sort of meeting or direct contact with sick people suffering from fever or cough or both
iii. **Managing a healthier lifestyle**
  Boosting up the immune system by having at least 7 h of sleep per night
  Controlling diet or maintaining regularity in taking sufficient amount of nutritious foods and fluids
  Making an effort to keep the blood glucose level under control
iv. **Utilizing feasible therapeutics and vaccines**
  **Chemoprophylaxis**
    Approval of drugs for pre-exposure and post-exposure chemoprophylaxis is not confirmed till now. Urgent randomized controlled trials should be conducted to attain proper evidence. Besides, chloroquine has exhibited antiviral activity against five known coronaviruses among seven, which includes the novel coronavirus SARS-CoV-2 [127]. Therefore, this drug is considered a superior candidate for prophylactic use [150]. Also, trials that are taking place in China have yielded promising preliminary findings [151], but the data are overall controversial. Some other trials are in progress: the PHYDRA trial (NCT04318015) and the COPCOV study (NCT04303507). Diabetic patients are also included in some of the studies. A clustered randomized clinical trial is also designed to evaluate the usage of lopinavir/ritonavir in post-exposure prophylaxis (NCT04321174).
  **Vaccines**
    A potent and secure vaccine would undoubtedly be preferable for high-risk individuals, such as those with cardiovascular disease (high blood pressure, history of heart attack, etc.), and diabetes and the aged ones. Therefore, different vaccines are being studied and investigated now: recombinant novel coronavirus vaccine (adenovirus type 5 vector) (NCT04313127) in the APICTH trial, mRNA-1273 vaccine (NCT04283461), and artificial antigen-presenting cells (AAPCs) as a vaccine (NCT04299724) [152].

#### Future guidelines

After the second world war, no other challenge is considered so tremendous as the COVID-19 pandemic. Identification of effective prevention and treatment strategies is a requisite of today’s world. The urgency of identifying adjuvant prevention as well as treatment strategies is needless to say. Deterioration of health has been observed in people with diabetes and comorbid patients, though the molecular and pathophysiology mechanisms lying behind this association are not yet completely clarified. The critical queries regarding precautionary measurements, management of COVID-19, and the protection of people with diabetes require transparent answers. Researchers, also the associated authorities, should take indispensable steps to find answers to these questions.

It is mandatory to set up standard case definitions, data collection, recording and sharing strategies, and operational instructions to allow easy interpretation and data comparison. Standardizing research protocols and pointing out research priorities indicate proper utilization of time and convenient resources. The function of pharmaceutical drugs required to prevent and treat COVID-19 according to their efficacy, safety, and cost convenience should be evaluated as a precedent. Besides, additional data are needed, especially observing the effects of both SGLT2 and ACEI/ARB's inhibitors in those who are infected and critically ill.

Strategies should be taken to detect cases and treatment purposes, provide care, and ensure the supply of necessary medicines for people with chronic diseases like diabetes. Doing so may diminish the risk of morbidity and mortality caused by such diseases through this harsh period. Again, utilizing human resources prudently in healthcare services and protecting their health is the demand of time. This contemporary challenge for healthcare systems should be an opportunity to upgrade service provision, receive learnings from successful regional and global strategies. Moreover, it is a resounding scope to take preparation for the upcoming enormous challenges of the world. However, the pandemic delineates the necessity of both care-for-all policies and combined public health measures.

## Conclusions

As discussed in the review, diabetic patients need scientific attention as they are a major risk group to manifest COVID-19. In addition, diabetic patients suffer from severe physical consequences from the virus rather than nondiabetic patients. More clinical cohort studies are necessary to conclude substantial proof for the relation between DM and elevated level of COVID-19 symptoms or higher mortality rate. However, the existing data are reliable enough to consider the diabetic community as a potential community largely under health threats during the COVID-19 pandemic. As DM patients already suffer from organ damage or decreased organ functionality, COVID-19 patients with DM must be treated accordingly in the hospital or household facilities. As a precaution, DM patients must maintain safety measurements in daily life to lower the chances of exposure to SARS-CoV-2.

Additionally, in case of virus contraction, the treating physician must consider the patient's prescribed DM medication regimen while treating COVID-19. During recovery, patients must follow the treatment regimen and maintain an ordered life. Furthermore, scientific research is required to identify if DM medication can hamper the COVID-19 treatment regimen to lower the mortality rate of COVID-19 patients with DM and ease the recovery process for the diabetic survivors.

## Data Availability

All the data are provided within the manuscript.

## Abbreviations

ROS: Reactive oxygen species
IgG: Immunoglobulin G
ACE2: Angiotensin-converting enzyme 2
